# Editorial: Recent advances in artificial intelligence-empowered ultrasound tissue characterization for disease diagnosis, intervention guidance, and therapy monitoring

**DOI:** 10.3389/fphys.2023.1234611

**Published:** 2023-06-28

**Authors:** Zhuhuang Zhou, Ke Lv, Po-Hsiang Tsui

**Affiliations:** ^1^ Department of Biomedical Engineering, Faculty of Environment and Life, Beijing University of Technology, Beijing, China; ^2^ Department of Ultrasound, Peking Union Medical College Hospital, Chinese Academy of Medical Sciences and Peking Union Medical College, Beijing, China; ^3^ Department of Medical Imaging and Radiological Sciences, College of Medicine, Chang Gung University, Taoyuan, Taiwan; ^4^ Institute for Radiological Research, Chang Gung University, Taoyuan, Taiwan; ^5^ Division of Pediatric Gastroenterology, Department of Pediatrics, Chang Gung Memorial Hospital at Linkou, Taoyuan, Taiwan

**Keywords:** ultrasound tissue characterization, deep learning, machine learning, artificial intelligence, ultrasound imaging

## 1 Introduction

Ultrasound tissue characterization involves investigating the interaction of ultrasound waves with biological tissues by analyzing ultrasound backscattered radiofrequency signals (quantitative ultrasound) and ultrasound images. In recent years, there has been a trend for enhancing ultrasound tissue characterization with artificial intelligence (AI), including machine learning and particularly deep learning techniques. These advanced data-driven models can assist in extracting “intelligently” more information from ultrasound backscattered radiofrequency signals and images. This Research Topic “*Recent advances in artificial intelligence-empowered ultrasound tissue characterization for disease diagnosis, intervention guidance, and therapy monitoring*” focuses on recent advances in AI-empowered ultrasound tissue characterization methods and techniques for improving disease diagnosis, intervention guidance, and therapy monitoring.

## 2 Inside the Research Topic

Shi et al. investigated the associations between electrocardiograms and carotid ultrasound parameters and explored the feasibility of assessing carotid health with electrocardiograms, using a cohort of healthy Chinese individuals (*n* = 319). The parameters analyzed were standard 12-lead electrocardiogram parameters such as the ST-segment amplitude, carotid ultrasound parameters of intima-media thickness and blood flow resistance index, as well as cardiovascular disease risk factors such as sex, age, and systolic blood pressure. Linear and stepwise multivariable regression models were conducted to investigate the associations between electrocardiograms and carotid ultrasound parameters. It was demonstrated that the ST-segment amplitude was associated with the resistance index of common carotid artery, indicating that electrocardiograms may be utilized to assess carotid health.

Jiang et al. combined AI methods to create a new spine ultrasound image segmentation model, ultrasound global guidance block network (UGBNet), providing a fully automatic and reliable spine segmentation and scoliosis visualization approach. A total of 102 adolescent idiopathic scoliosis patients were included, with approximately 2000 B-mode ultrasound images and corresponding spatial data collected from each patient. The UGBNet incorporated a global guidance block module that integrated spatial and channel attention, through which long-range feature dependencies and contextual scale information could be learnt. The performance of the UGBNet model in semantic segmentation on spinal ultrasound datasets was evaluated and compared with several classical learning-based segmentation methods, such as U-Net. The experimental results demonstrated the superiority of the UGBNet, with a Dice score of 74.2%.

Bai et al. proposed a framework for evaluating fetal descent with transperineal ultrasound images, including image segmentation, target fitting, and angle of progression calculation (*n* = 313). For image segmentation, a double branch segmentation network (DBSN) was presented, which consisted of two parts: an encoding part receiving image input, and a decoding part composed of deformable convolutional blocks and conventional convolutional blocks. The decoding part included lower and upper branches, and the feature map of the lower branch was used as the input of the upper branch to assist the upper branch in decoding after being constrained by the attention gate. For a transperineal ultrasound image, areas of the pubic symphysis and fetal head were segmented with the DBSN, and the ellipse contours of segmented regions were fitted with the least square method. Three endpoints were determined for calculating angles of progression. Five-fold cross-validations were used for model evaluation, with comparisons to four state-of-the-art methods. Their method achieved the highest Dice score (93.4%), the smallest average surface distance (6.268 pixels) and the lowest angle of progression difference (5.993°). Similar performance (Dice: 91.7%, average surface distance: 7.729 pixels: angle of progression difference: 5.110°) was obtained on a public dataset with >3,700 transperineal ultrasound images.

Yu et al. investigated the potential of combining B-mode ultrasound image features with shear wave elastography features to improve the classification of non-specific low back pain patients (*n* = 52). B-mode ultrasound images and shear wave elastography data from multiple sites were collected. The visual analogue scale was used as the reference standard for classification. Image features were extracted and selected from the data and a support vector machine model was used for classification. Five-fold cross-validations were used for model evaluation, and the accuracy, precision, and sensitivity were calculated. An optimal feature set of 48 features were obtained, among which the elasticity feature of shear wave elastography had the most significant contribution to the classification task. The accuracy, precision, and sensitivity of 0.85, 0.89, and 0.86 were obtained.

Tian et al. proposed to upgrade a Siamese architecture-based neural network for robust and accurate landmark tracking in contrast-enhanced ultrasound videos. They introduced two modules into the original Siamese architecture. A temporal motion attention based on Lucas Kanade optic flow and Karman filter was employed to model the regular movement and better instruct location prediction. In addition, a pipeline of template update was incorporated to provide timely adaptation to feature changes. Their method yielded an average mean intersection over union of 86.43% on 33 labeled videos with a total of 37,549 frames. Compared to other classical tracking models, their model had a smaller tracking error of 19.2 pixels and a root mean square error of 27.6 with a frame rate of (8.36 ± 3.23) frames per second.

## 3 Summary

A major issue of conventional ultrasound tissue characterization methods without AI is about the manner they extract information from ultrasound backscattered radiofrequency signals and images. Such a manner can be either single-purpose or single-level. For instance, B-mode imaging extracts the log-compressed envelope amplitude information exclusively, while ultrasound attenuation, elastography, or Nakagami imaging extract the information of acoustic attenuation, tissue elasticity (or time delay), Nakagami parameter, respectively, using a specific mathematical or physical model. In contrast, AI-empowered ultrasound tissue characterization methods use data-driven models to learn more abundant and multi-level information from ultrasound backscattered radiofrequency signals and images. Such learnt information can help improve object detection, disease diagnosis, intervention guidance, and therapy monitoring. For instance, the deep learning models trained on ultrasound backscattered signals have shown great potential in clinical fatty liver characterization. We believe that incorporating AI into biomedical ultrasound will represent a new direction for ultrasound tissue characterization, which is a growing interest.

